# Commentary: Gene Therapy: A Promising Approach for Neuroprotection in Parkinson's Disease?

**DOI:** 10.3389/fnana.2017.00040

**Published:** 2017-05-19

**Authors:** Juan Segura-Aguilar

**Affiliations:** Molecular and Clinical Pharmacology, Faculty of Medicine, Institute of Biomedical Sciences (ICBM), University of ChileSantiago, Chile

**Keywords:** aminochrome, dopamine oxidation, neurodegeneration, astrocytes, dopaminergic neurons

This review discusses the possible use and effectiveness of gene therapy in Parkinson's disease (PD) (Valdés and Schneider, 2016). This is an excellent critical review about the possibilities of developing a successful gene therapy to provide neuroprotection in PD. The review analyzes potential targets for gene therapy such as: (i) genes associated with familial forms of the disease such as alpha-synuclein that aggregates to neurotoxic oligomers, or genes with loss-of-function mutations involved in recessive forms of the familial forms of the disease (Parkin and PINK1). However, both the silencing of the alpha-synuclein gene and the long-term chronic overexpression of Parkin and PINK1 induce degeneration in the nigrostriatal system, and the question is, whether this gene therapy can help patients with sporadic Parkinson's disease. (ii) Genes associated with autophagy such as the lysosomal enzyme β-glucocerebrosidase, and the transcription factor TFEB which is involved in the regulation of lysosomal expression and the regulation network. (iii) Peroxisome proliferator-activated receptor gamma coactivator 1-alpha (PGC-1α), a master regulator of mitochondrial biogenesis. PGC-1α activity exhibits direct interaction, and the alpha-synuclein pathology and Parkin control PGC-1α expression. (iv) Genes related to endoplasmic reticulum stress including GRP78/BiP and IRE1α. (v) Neurotrophic factors such as mesencephalic astrocyte-derived neurotrophic factor and cerebral dopamine neurotrophic factor. However, clinical studies done with neurturin, a member of the GDNF family, showed no benefit for PD patients (Valdés and Schneider, 2016).

A successful gene therapy has to point to what triggers these mechanisms in the disease in order to halt the progression. In the Parkinsonism types associated with metals, pesticides, neuroleptics, or other factors we know what induces these forms of the disease. We know that specific mutations induce the familial forms of the disease and that gene therapy can be an option for these forms. However, what induces the degeneration of dopaminergic neurons containing neuromelanin in the idiopathic form of the disease is still unknown. The discovery of genes associated with a familial form of the disease represents an enormous advance in basic research undertaken in order to understand the role of these proteins in the loss of dopaminergic neurons in the nigrostriatal system. There is a general consensus in the scientific community that the formation of neurotoxic oligomers of alpha-synuclein, mitochondria dysfunction, protein degradation dysfunction, neuroinflammation, and oxidative and endoplasmic reticulum stress are all involved in the loss of nigrostriatal dopaminergic neurons (Segura-Aguilar et al., 2014, 2016). However, many of these mechanisms are part of a vicious circle, which eventually leads to neurodegeneration.

Another possible candidate for triggering dopaminergic neuron degeneration in the nigrostriatal system is aminochrome. Aminochrome is an *o*-quinone formed during the oxidation of dopamine to neuromelanin within dopaminergic neurons which are lost during the degenerative process associated with Parkinson's disease. Aminochrome induces: (i) mitochondrial dysfunction, (ii) dysfunction of protein degradation, (iii) the formation of neurotoxic oligomers of alpha-synuclein, (iv) oxidative stress, (v) endoplasmic reticulum stress and (vi) neuroinflammation (Santos et al., 2017) (Figure 1B). However, aminochrome can also participate in neuroprotective reactions. There are two neuroprotective reactions which allow the oxidation of dopamine to *o*-quinones, resulting in the formation of the pigment neuromelanin. The first of these is the two-electron reduction of aminochrome to leukoaminochrome, catalyzed by DT-diaphorase (Figure 1A). This enzyme is a unique flavoenzyme that catalyzes the two-electron transfer reduction of quinones to hydroquinones, which is expressed in both dopaminergic neurons and astrocytes in the substantia nigra. DT-diaphorase prevents aminochrome-induced cell death, mitochondrial dysfunction, oxidative stress, proteasomal system dysfunction, autophagy dysfunction, α- and β-tubulin aggregation (required for the fusion of autophagosomes and lysosomes), and the formation of neurotoxic alpha-synuclein oligomers (Segura-Aguilar et al., 2014). It has been suggested that the formation of alpha-synuclein oligomers during dopamine oxidation to *o*-quinones is mainly dependent on non-covalent interactions between alpha-synuclein and these *o*-quinones (Bisaglia et al., 2010). Interestingly, DT-diaphorase prevents alpha-synuclein oligomers' neurotoxicity (Muñoz et al., 2015). The second neuroprotective reaction involves the conjugation of aminochrome with GSH (Figure 1A). Glutathione transferase M2-2 (GSTM2) catalyzes the conjugation of GSH and aminochrome to 4-S-glutathionyl-5,6-dihydroxyindoline, which is stable under biological oxidizing conditions as it cannot be oxidized by hydrogen peroxide, superoxide or oxygen. GSTM2 also catalyzes the conjugation of GSH with dopamine *o*-quinone to form 5-glutathionyl dopamine, and the degradation product of this conjugate, 5-cysteinyl dopamine, has been detected in the neuromelanin, substantia nigra, and cerebrospinal fluid of PD patients. GSTM2 is only expressed in astrocytes and prevents aminochrome-induced cell death, autophagy, and lysosome dysfunction in an astrocyte cell-line model (U373MG cells). However, GSTM2 also plays a protective role against aminochrome-induced cell death in dopaminergic neurons. U373MG cells secrete GSTM2 into the conditioned medium of differentiated SH-SY5Y cells, which then take up GSTM2 from the conditioned medium, thus preventing aminochrome-induced cell death in the SH-SY5Y cells (Cuevas et al., 2015; Segura-Aguilar et al., 2016; Figure 1A). Both of these protective reactions against aminochrome neurotoxicity can be a target for gene therapy in the sporadic form of PD. The prevention of aminochrome-induced neurotoxic effects is essential to halt the progression of the disease because: (i) aminochrome induces mitochondrial dysfunction, the formation of neurotoxic oligomers of alpha-synuclein, dysfunction of protein degradation, and oxidative and endoplasmic reticulum stress and (ii) aminochrome is formed inside dopaminergic neurons lost during the disease (Figure 1B).

**Figure 1:**
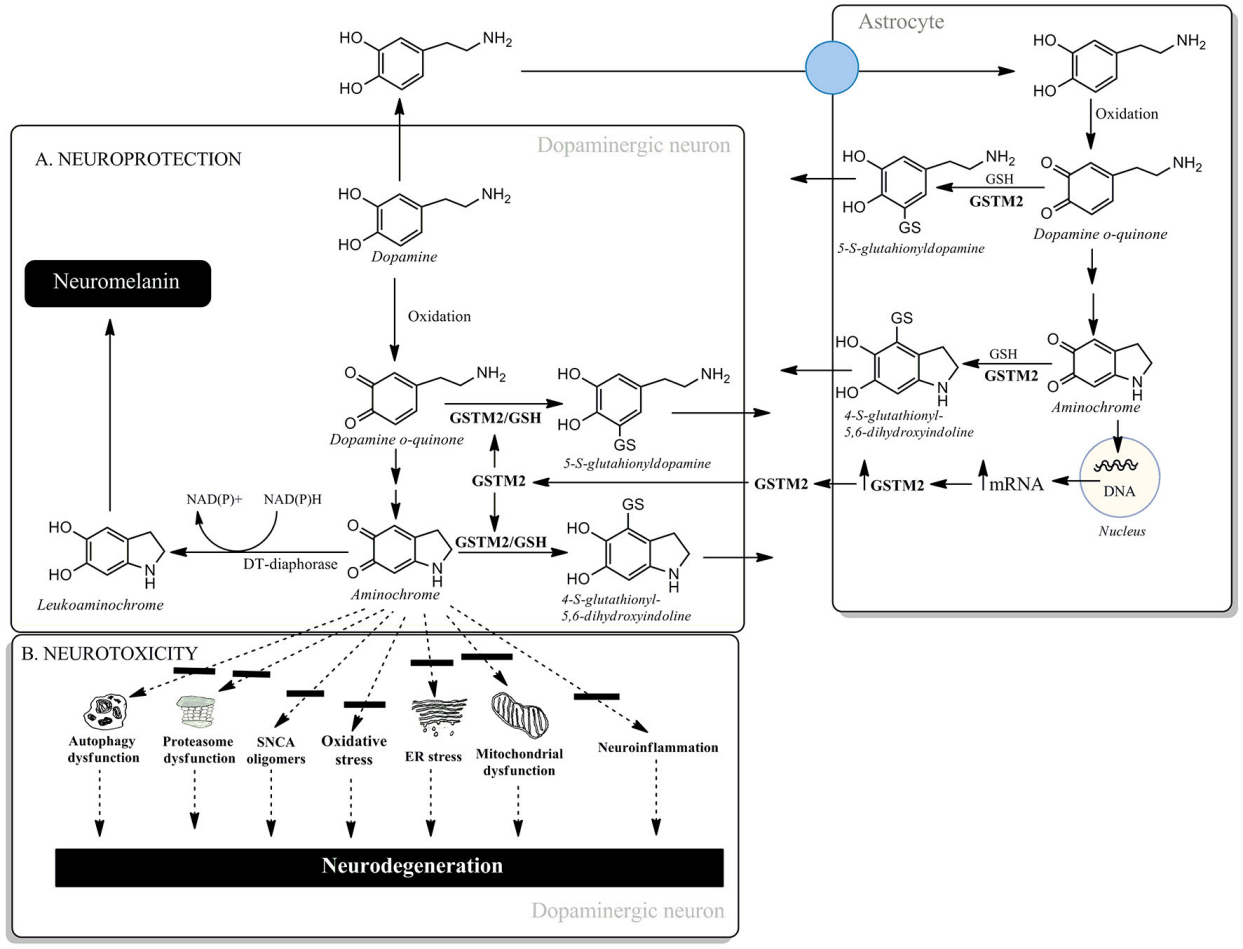
**(A)** Neuroprotective and **(B)** neurotoxic mechanisms of aminochrome.

In conclusion, the use of adeno-associated virus vectors (AVV) in various clinical studies has shown that the use of these vectors is safe and efficient. Different gene therapies are in progress for both the familial and sporadic forms of Parkinson's disease. The aims of these therapies for the familial form of the disease include the reduction of the levels of SNCA and the expression of Parkin. For sporadic Parkinson's these proposals are focused on the dysfunction of autophagy (AAV-GBA-1 and AAV-Beclin-1), mitochondrial dysfunction (AAV-PGC-1a), endoplasmic reticulum (AAV-XBP1s and AAV-GRP78/Bip), neurotrophic support (AAV-neurturin, AAV-GDNF, AAV-MANF, and AAV-CDNF), and aminochrome neurotoxicity during dopamine oxidation to neuromelanin (AAV-DT-diaphorase and AAV-GSTM2). It seems plausible that a new possible target for gene therapy will continue to increase in the next years.

## Author contributions

The author confirms being the sole contributor of this work and approved it for publication supported by FONDECYT 1170033.

### Conflict of interest statement

The author declares that the research was conducted in the absence of any commercial or financial relationships that could be construed as a potential conflict of interest. The reviewer MS and handling Editor declared their shared affiliation, and the handling Editor states that the process nevertheless met the standards of a fair and objective review.
